# 

**Published:** 1896-09

**Authors:** 


### Ohio State Dental Society.

The annual meeting of this society will be held in Columbus, on Tuesday, December 1, 1896. It is very desirable that there be a large attendance, and it is to be hoped that there will be a large addition to the membership. In a State as large as Ohio, and with about one thousand dentists, and with the history that the profession in the State has, there ought, certainly, to be half of that number in the society. Why are they not there? Who can tell? Can any of the habitual absentees frame an excuse that is satisfactory to themselves? Let every one try it. Sometimes sickness of one's self, or his family, may be a valid excuse for absence, but a very small percent of the whole will suffice for this class of absentees. Another excuse is, "I never learn anything at these meetings." Well, there may be sometimes grounds for this excuse; one may be so ignorant and dull as not to be able to learn any thing; well, of course, such a one should be excused. Another says, if not in words, by his manner, that he "knows about all that is worth knowing of professional matters." Well, that usually means that he has a small capacity and is not able to hold much, and it is already full. Well, that being true, he might be excused, and especially if he was not able to give up any thing he has; but, then, might not some one pump something out of him even if it should be but air. Well, this variety of dentist is not very numerous, indeed, is rather rare, and may, without much loss, be excused.

Another says, "Well, I can not really afford the loss of so much time and money."

Well, dear sir, you can not afford to be absent, even if you have to walk to the meeting and carry your lunch-basket. Just think what you will miss! In the first place, you will lose the acquaintance of many of your professional brethren, and the social influence of such acquaintance; a knowledge of our truly professional brothers is an acquisition not to be lightly esteemed—it is one of the elements of success. And further, no aspiring dentist ever attends a dental meeting that he does not gain something, and usually much of practical matters and of of scientific as well, if he brings something to carry it in. So come on, brother, and bring the biggest basket you have, and you need never go away empty; and it will be all the better if you bring something in the basket. We are sometimes surprised to find how much we can help those whom we have supposed to be more favored than ourselves in attainments, and, indeed, it must be remembered that many a time our efforts are most helpful when we think but little of them. So let the weak help the strong.

Another class, which is too common, consists of those who have no care or interest in their profession outside of their personal affairs; they have not learned that they can be helped or benefited by association with their fellows; to such we would suggest—you had better think over this subject seriously, and see if you have not been self-deceived; note the fact that those who have any good degree of attainments and desirable standing in the profession, and with those outside, too, are those who have been active in association work. Think over these things, and then act upon the convictions you may have. Let there be a great outpouring of the dentists of Ohio at the coming State Society meeting.

### CONTENTS.

COMMUNICATIONS.

### THE DENTAL REGISTER,

**A MONTHLY MAGAZINE DEVOTED TO THE INTERESTS OF THE**

### DENTAL PROFESSION.

*Terms Reduced to* $2.00 *Per Year. Single Copies,* 25 *Cents.*

**EDITED BY PROF. J. TAFT, D.D.S.**

—AND—

**Published by SAM'L A. CROCKER & CO., Ohio Dental and Surgical Depot.**

The volume commences in January and closes in December.

The REGISTER being issued monthly is always fresh, therefore Indispensable to the practitioner who desires to keep posted up with the new inventions and improvements which are daily developed. Neither expense nor effort will be spared to give value and variety to its contents. Articles will be illustrated with well executed engravings whenever they will add to the interest or assist to explanation.

Its extensive circulation and frequency of issue make it one of the BEST DENTAL ADVERTISING MEDIUMS in the United States.

All subscriptions must begin with January or July.

Local or special notices, five lines or less, will be inserted for $3 each insertion; over five lines, 50 cts. per line.

All advertising bills payable in advance, or at furthest, after the issue of the first number containing the advertisement. All transient advertisements to be paid for in advance.

☞CONTRIBUTIONS SOLICITED.

Exclusively Editorial Communications should be addressed to the Editor.

The latest number of the REGISTER may be ordered with or without other goods at any time, and all letters should be addres[illegible]

